# Interstitial null-distance time-domain diffuse optical spectroscopy using a superconducting nanowire detector

**DOI:** 10.1117/1.JBO.28.12.121202

**Published:** 2023-04-03

**Authors:** Vamshi Damagatla, Pranav Lanka, Annalisa Brodu, Niels Noordzij, Jessie Qin-Dregely, Andrea Farina, Antonio Pifferi

**Affiliations:** a Politecnico di Milano, Dipartimento di Fisica, Milano, Italy; b Tyndall National Institute, Biophotonics, IPIC, Cork, Ireland; c Single Quantum BV, Delft, The Netherlands; d Istituto di Fotonica e Nanotecnologie, Consiglio Nazionale delle Ricerche, Milano, Italy

**Keywords:** diffuse optics, time domain, biophotonics, interstitial fiber, spectroscopy, superconducting nanowire detector

## Abstract

**Significance:**

Interstitial fiber-based spectroscopy is gaining interest for real-time *in vivo* optical biopsies, endoscopic interventions, and local monitoring of therapy. Different from other photonics approaches, time-domain diffuse optical spectroscopy (TD-DOS) can probe the tissue at a few cm distance from the fiber tip and disentangle absorption from the scattering properties. Nevertheless, the signal detected at a short distance from the source is strongly dominated by the photons arriving early at the detector, thus hampering the possibility of resolving late photons, which are rich in information about depth and absorption.

**Aim:**

To fully benefit from the null-distance approach, a detector with an extremely high dynamic range is required to effectively collect the late photons; the goal of our paper is to test its feasibility to perform TD-DOS measurements at null source–detector separations (NSDS).

**Approach:**

In particular, we demonstrate the use of a superconducting nanowire single photon detector (SNSPD) to perform TD-DOS at almost NSDS (≈150  μm) by exploiting the high dynamic range and temporal resolution of the SNSPD to extract late arriving, deep-traveling photons from the burst of early photons.

**Results:**

This approach was demonstrated both on Monte Carlo simulations and on phantom measurements, achieving an accuracy in the retrieval of the water spectrum of better than 15%, spanning almost two decades of absorption change in the 700- to 1100-nm range. Additionally, we show that, for interstitial measurements at null source–detector distance, the scattering coefficient has a negligible effect on late photons, easing the retrieval of the absorption coefficient.

**Conclusions:**

Utilizing the SNSPD, broadband TD-DOS measurements were performed to successfully retrieve the absorption spectra of the liquid phantoms. Although the SNSPD has certain drawbacks for use in a clinical system, it is an emerging field with research progressing rapidly, and this makes the SNSPD a viable option and a good solution for future research in needle guided time-domain interstitial fiber spectroscopy.

## 1 Introduction

The use of interstitial fiber spectroscopy (IFS) for medical applications and as a guidance tool in minimally invasive *in vivo* surgical procedures is an attractive area of interest for research.^1^^–^^3^ With the use of needle-guided optical fibers and the integration with existing imaging probes,^4^ it has the potential to support with functional volumetric assessment in endoscopic and operative procedures,^5^ perform needle-based “optical biopsies,” aid in characterization and *in vivo* monitoring of photodynamic therapy,^2^^,^^6^ assist in innovative real time drug delivery monitoring systems, and in general, help in the shift from open chest and open cranial to minimally invasive surgery.

A plethora of photonics techniques have been proposed and investigated to provide *in vivo* diagnostics from inside the human body, exploiting different light–matter interactions, such as reflection, absorption, scattering^7^—both elastic and inelastic (e.g., Raman)—, and fluorescence.^8^ Further, functionalization of the fiber through plasmonics or Bragg gratings can provide additional information from the tissue.^9^ In most cases, these techniques are point-based and provide information from a small region around the fiber tip or the fiber itself. In this context, time-domain diffuse optical spectroscopy (TD-DOS)^10^^–^^18^—which is based on the detection of the distribution of time-of-flight (TOF) of photons traveling into the tissue—offers several advantages: (i) the capability to disentangle the absorption ($\mu_{a}$) and reduced scattering ($\mu_{s}^{\prime }$) coefficients; (ii) an extended probed depth in the tissue (a few cm) related to the photon TOF; and (iii) insensitivity to amplitude fluctuations due, for instance, to optical coupling or bleeding.

Usually, in TD-DOS, the source and detector fibers are separated by a few cm to collect strongly diffused photons. Conversely, for IFS, a single needle with either a single fiber^19^ or two fibers adjacent to each other helps in reducing the invasiveness of the probe, but this stipulates the adoption of the null source–detector separation (NSDS) approach. It has been shown theoretically and experimentally that in TD-DOS the mean path length traveled and mean depth reached are both independent of the source–detector distance (ρ),^20^^,^^21^ and hence, it is sufficient to either only “collect” or “select” photons with longer TOF (late photons) to have information about the penetration depth and the absorption. Although NSDS has the advantages of better contrast, spatial resolution, and increased signal,^22^ it has the drawback of an overwhelming number of early photons that outnumber the late photons by many orders of magnitude and saturate the standard detectors, such as silicon photomultipliers (SiPM), which have long diffusion tails that reduce their dynamic range and make it difficult to distinguish the small number of late arriving photons.^23^ This limitation has been since overcome by the use of ultrafast gated single photon avalanche diodes (SPADs) to reject the burst of early photons, and the application of high-dynamic-range TOF spectroscopy has been demonstrated using a single fiber,^19^ as well as at small (mm) source–detector separations, for use in functional imaging of brain activation^24^ and noncontact diffuse optical imaging.^25^^–^^27^ However, the SPAD has a limitation of smaller detector area,^28^ and gating of the SPAD results in a new background effect—the memory effect (ME), attributed to the large number of photons impinging on the detector before the opening of the gate.^29^ This ME adds a decaying tail to the background noise and has been seen as the limiting factor in using fast gated SPADs.^30^^,^^31^

Here we propose an alternate approach based on the use of a detector with a high dynamic range and narrow temporal response to sustain the burst of early photons at NSDS even without the adoption of gating methods. In particular, we exploit a superconducting nanowire single photon detector (SNSPD) offering very high temporal resolution (<20  ps) and detection efficiency (>85%) in the near-infrared (600 to 1100 nm), coupled with very low background noise (dark count rate <10  Hz), and the absence of temporal decay tails or ME.

We provide a proof-of-concept of this approach in a simple scenario of two adjacent fibers immersed in a homogeneous medium, validating the technique both on Monte Carlo (MC) simulations and on measurements using realistic tissue phantoms. As additional information, we demonstrate that in the NSDS configuration and for $\mu_{s}^{\prime }$ around $10\;{\mathrm{cm}}^{-1}$ or more, the temporal shape of photons’ TOF is independent of $\mu_{s}^{\prime }$, permitting an easier assessment of $\mu_{a}$.

The paper is structured as follows: after a theory section developing the peculiarities of the diffusion equation (DE) for the interstitial NSDS geometry, we present the experimental and analytical methods used in this study. Then the validity of the approach and the independence from $\mu_{s}^{\prime }$ is tested against MC simulations. Finally, TD-DOS measurements over the 600–1100 nm range using adjacent optical fibers immersed in a liquid phantom are discussed to ascertain the applicability of the technique.

## 2 Theory

The radiative transfer equation is simplified using a $P_{1}$ approximation of its series expansion to obtain the DE [Eq. (1)] for the fluence (ϕ), where $q_{0}(\overset{\to }{r},t)$ is an isotropic source:

$$(\frac{\partial }{v\partial t}-D\nabla^{2}+\mu_{a})\phi (\overset{\to }{r},t)=q_{0}(\overset{\to }{r},t).$$ (1)

We present our case primarily for an infinite medium to allow for simplification and to accommodate our goal of performing interstitial measurements, which require detection of photons/fluence inside the bulk of the medium, a point that other commonly used geometrical configurations permit only on the boundaries. For the case of an infinite homogeneous medium, the time-dependent Green function for the fluence is given by Eq. (2), with $D=1/3\mu_{s}^{\prime }$ defined as the diffusion coefficient:

$$\phi (\overset{\to }{r},t)=\frac{v}{{\left(4\pi Dvt\right)}^{3/2}}\;\exp(-\frac{r^{2}}{4Dvt})\exp(-\mu_{a}vt).$$ (2)

From the diffusion approximation, we know that the DE is valid only for “nearly” isotropic radiance and “slow” time variations of the flux inside the medium, which are satisfied when the photons have undergone many scattering events or have traveled at least a distance of $l^{\prime }=1/\mu_{s}^{\prime }$, defined as the “transport mean free path,” which is the mean distance traversed by the photons before they lose information about their initial direction. This requires the scattering effects of the volume to be predominant over absorption.^32^ For a standard case of $\mu_{s}^{\prime }=10\;{\mathrm{cm}}^{-1}$, $l^{\prime }$, the minimum distance, turns out to be 1 mm, which is easily satisfied by commonly used source–detector separations. However, at very small distances and more importantly at NSDS, with ρ=0, this condition necessitates that we must only select photons with longer paths or, in other words, later arrival times. Further examining the expression for the fluence at ρ=0, we observe that the time-dependent relation of ϕ on $\mu_{s}^{\prime }$ is lost, that is,

$$\phi (\overset{\to }{r},t)=\frac{v}{(4\pi Dv{)}^{3/2}}t^{-3/2}\;\exp(-\mu_{a}vt).$$ (3)

The first time-independent factor can be neglected when analyzing TD-DOS data because typically only the temporal shape is considered, leaving the amplitude term generally unknown.^33^ Therefore, under DE and the NSDS for an infinite medium, there is no temporal dependence of ϕ on $\mu_{s}^{\prime }$. As a consequence, the fitting of the experimental data could be eased because only 1 free parameter ($\mu_{a}$) is left. Surely, when the DE approximation is no longer valid, e.g., at early times, or for not-null ρ, the above simplification fails. The validity of this simplified Eq. (3) will be tested against MC simulations in Sec. 3.3.

## 3 Methodology

### 3.1 Experimental Setup

**Fig. 1:**
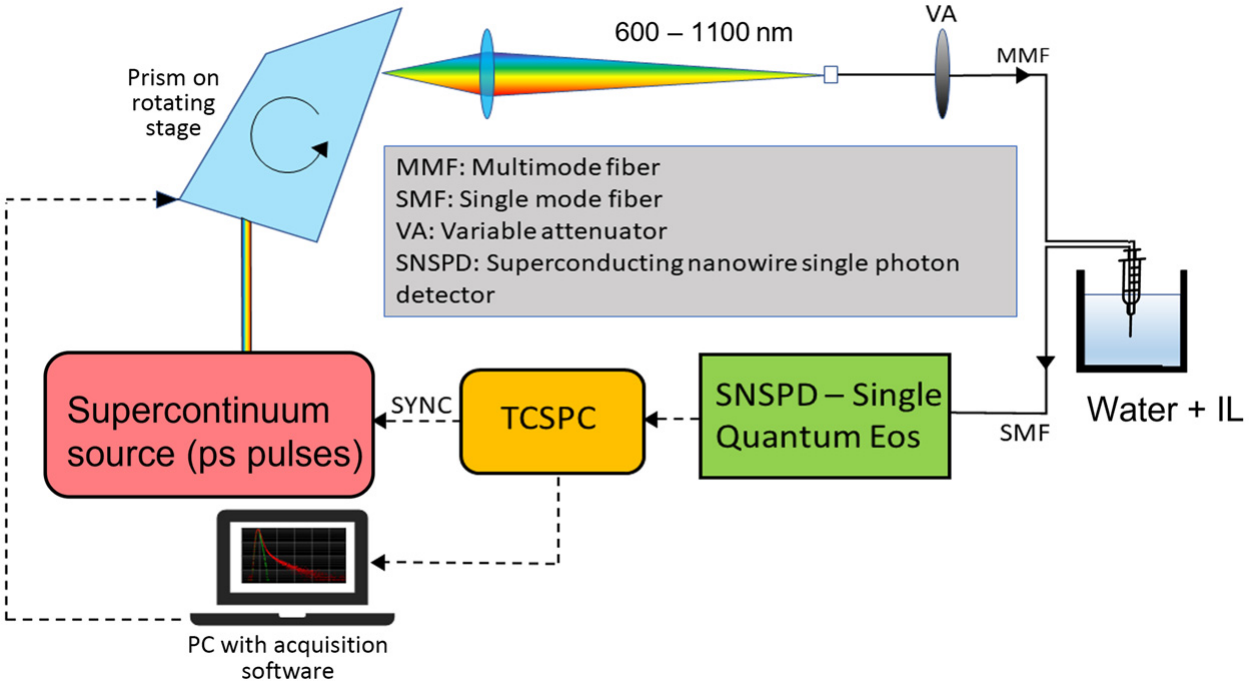
Schematic of the experimental setup.

The experiments were performed in a setup similar to a state-of-the-art TD-DOS system^34^ with a variation in the use of a different detection stage (Fig. 1). Picosecond pulses at 80 MHz repetition rate were generated using a supercontinuum laser (SuperK Extreme, NKT Photonics, Denmark) and were spatially dispersed using a Pellin Broca prism mounted on a rotating stage. This helped with obtaining the spectrum from 600 to 1100 nm in steps of 20 nm, with the linewidth varying from 3 nm at 650 nm to 7 nm at 1100 nm. These pulses were then coupled into a 50/125-μm graded index optical fiber to be incident into the sample after passing through a variable attenuator. The diffusely reflected photons were collected through a single-mode fiber and detected by a four-channel SNSPD (Eos, Single Quantum, Netherlands). The acquired signal was processed with a time correlated single photon counting module (SPC-130, Becker and Hickl, Germany) to obtain distributed time-of-flight (DTOF) curves. The probe used in this setup was made of bare fibers with their protective sheaths removed at the ends to get as close to the NSDS scenario as possible. The instrument response function (IRF) was taken in a tip-to-tip geometry setup along with external shielding to prevent leakage of external light into the signal.

### 3.2 Sample Preparation

Experiments were performed on liquid phantoms made of calibrated aqueous dilutions of Intralipid (IL) and ink to get the desired optical properties.^35^ The IL-based phantoms had a volume of 995 ml and were housed in a black PVC tank (absorbing boundaries) of dimensions 10×7×14  cm (l×b×h); the fibers were inserted to a depth of ≈3  cm. Three kinds of measurements were performed: (1) to test the linearity of the system;^36^ (2) to test the system accuracy in the retrieval of the water spectrum; and (3) to study the effect of variation of $\mu_{s}^{\prime }$ on the recovered $\mu_{a}$. For recovering the water spectrum, the liquid phantom was prepared with no added ink and a nominal value of $\mu_{s}^{\prime }=10\;{\mathrm{cm}}^{-1}$ at 750 nm, whereas for the linearity measurements, $\mu_{a}$ was varied by adding calibrated ink quantities while keeping $\mu_{s}^{\prime }$ fixed as for the previous case. Finally, to test the effects of variation of $\mu_{s}^{\prime }$, the ratio of IL in water was varied to get values of $\mu_{s}^{\prime }=5,10,15,20\;{\mathrm{cm}}^{-1}$ at 750 nm.

### 3.3 Simulations

MC simulations were performed using the open source light transport simulator software —Monte Carlo eXtreme (MCX).^37^ A discretized volume of 100×100×70  voxels with a voxel size of $1\;{\mathrm{mm}}^{2}$ and “completely absorbing” boundary condition (BC) on all boundaries was employed. The anisotropy coefficent was set at g=0.89 along with $\mu_{s}=90.9\;{\mathrm{cm}}^{-1}$ to obtain a reduced scattering coefficient of $\mu_{s}^{\prime }=10\;{\mathrm{cm}}^{-1}$. These optical parameters were kept the same throughout the volume to get a homogenous medium. Absorption was added later using the principle of the Beer–Lambert law. A light source with a pencil beam profile and a detector with a radius of 1 mm were both placed at a voxel position of (50, 50, 20) to replicate the NSDS scenario in an infinite medium, and the simulations were performed for a total run time of 3 ns with a 3-ps timestep. A total of $10^{6}$ photons were input into the medium, and the path trajectories of the detected photons and the fluence in every voxel were recorded. The process was repeated for four different values of $\mu_{s}^{\prime }=5,10,15,20\;{\mathrm{cm}}^{-1}$, and for each $\mu_{s}^{\prime }$, 10 simulations with random initial seeds were run to also study the random error in the process. Due to the “infinite medium” model being used for analysis, the fluence output was preferred over photon counts, and hence, the fluence was plotted as a function of time to obtain the DTOF curves. For each simulated curve, absorption was added using the Beer–Lambert law to get 16 DTOFs with increasing absorption values, equally spaced on a logarithmic scale, with $\mu_{a}$ in the range of $\mu_{a}=0.001$ to $1\;{\mathrm{cm}}^{-1}$. Four different IRFs were used to study the effect of system: (1) delta function (2) Gaussian IRF with FWHM = 75 ps, (3) IRF of the system with a SiPM detector,^23^ and (4) IRF of the system with an SNSPD. The DTOFs were normalized with respect to the area under the curve and convolved with similarly normalized IRFs, and finally, Poisson noise was added to the curves.

**Fig. 2:**
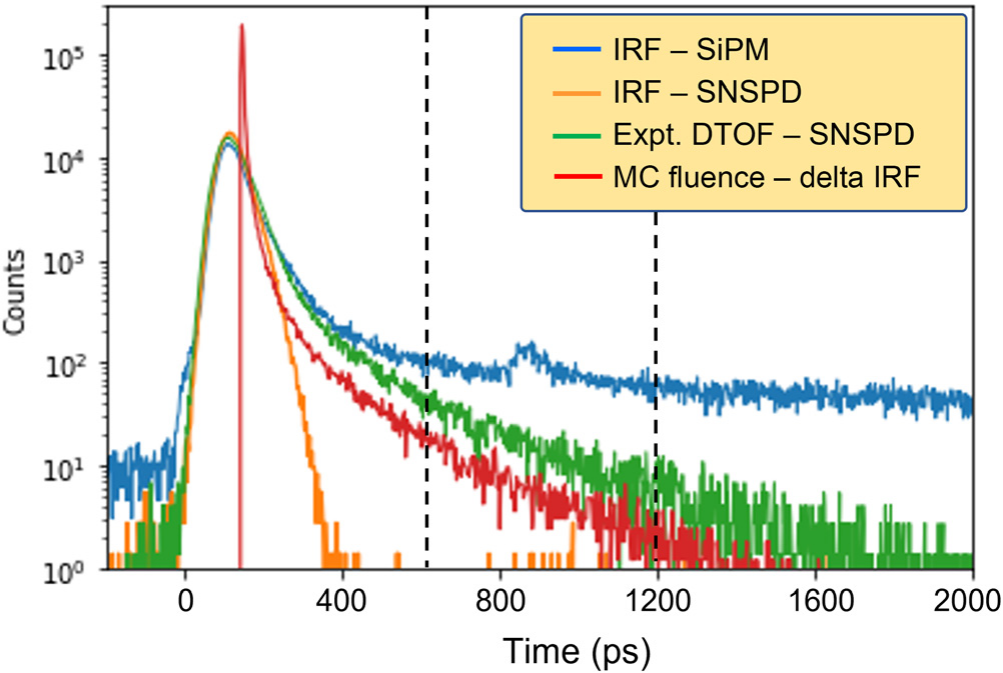
Comparision of the IRFs of the SNSPD and SiPM with the MC fluence and experimental DTOF (SNSPD) at $\mu_{a}=0.01\;{\mathrm{cm}}^{-1}$ and $\mu_{s}^{\prime }=10\;{\mathrm{cm}}^{-1}$.

Figure 2 shows an example of the IRF of the setup at 700 nm, as measured with an SNSPD (orange) and a SiPM (blue). The SNSPD IRF has an almost Gaussian appearance with extremely low noise characteristics of ≈140 counts over 2500 channels compared with a peak value of ≈13,500 counts (1 s acquisition time), yielding a very high dynamic range of >55  dB in the case of our measurements. The red curve represents an MC simulated fluence, and the green curve gives the DTOF of a measurement on a liquid phantom with nominal $\mu_{a}=0.01$ and $\mu_{s}^{\prime }=10\;{\mathrm{cm}}^{-1}$. As is evident, the peak of the DTOF corresponds exactly with the IRF, thus making it impossible to retrieve any information from it using an inverse procedure. Hence the fitting range is completely on the tail of the curve.

### 3.4 Analysis

For the analysis, we decided to use the infinite medium model, despite having finite boundaries. However, because the simulations have a maximum photon TOF of 3 ns, the boundaries at 50 mm are hardly reached.^21^ The absorption properties of the system were then retrieved using an inversion procedure, in which the analytical solution of the DE for the infinite medium is convolved with the IRF and is fitted to the experimental/simulated data curves using the Levenberg–Marquardt algorithm for nonlinear optimization.^38^ Generally, the freely varying parameters $\left(\mu_{a},\mu_{s}^{\prime }\right)$ and the fitting range are varied and optimized to obtain the best fits. In this case, as discussed in Sec. 2, $\mu_{s}^{\prime }$ was kept constant, and both $\mu_{a}$ and the fitting range were varied to recover only the absorption values. The fitting range is considered to be a fraction of the peak count on the rising edge (“−” sign) and falling edge (“+” sign), and a sample fitting range is shown in Fig. 2 with the black dotted lines representing the beginning and end at +0.1% and $+10^{-3}\%$ of the peak, respectively. In particular, we varied the left edge of the fitting range from −80% to $+10^{-3}\%$ (thus overcoming the peak) while fixing the right edge to $+10^{-4}\%$.

## 4 Simulations

**Fig. 3:**
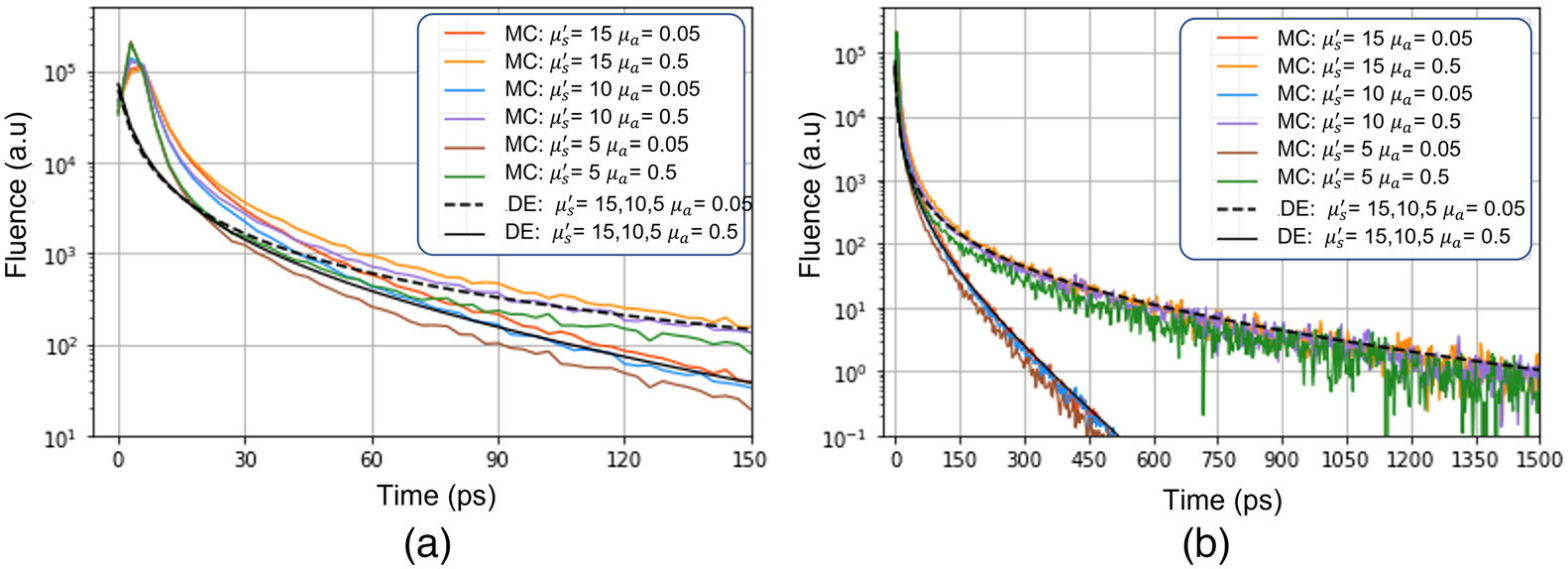
MC simulations (solid colored lines), and analytical solutions of the DE (black lines). (a), (b) The same curves but at shorter and longer time scales, respectively.

Figure 3 shows a comparison of MC simulations with the Green’s function derived from the DE. The key mismatch between the DE (dashed lines) and MC (solid lines) occurs at early times, when the DE approximation fails, whereas at longer times, there is a good agreement on the slope between the two models. This implies that, as long as only the late photons are selected, we can attempt to fit the MC generated DTOFs, and the experimental measurements to the DE.

**Fig. 4:**
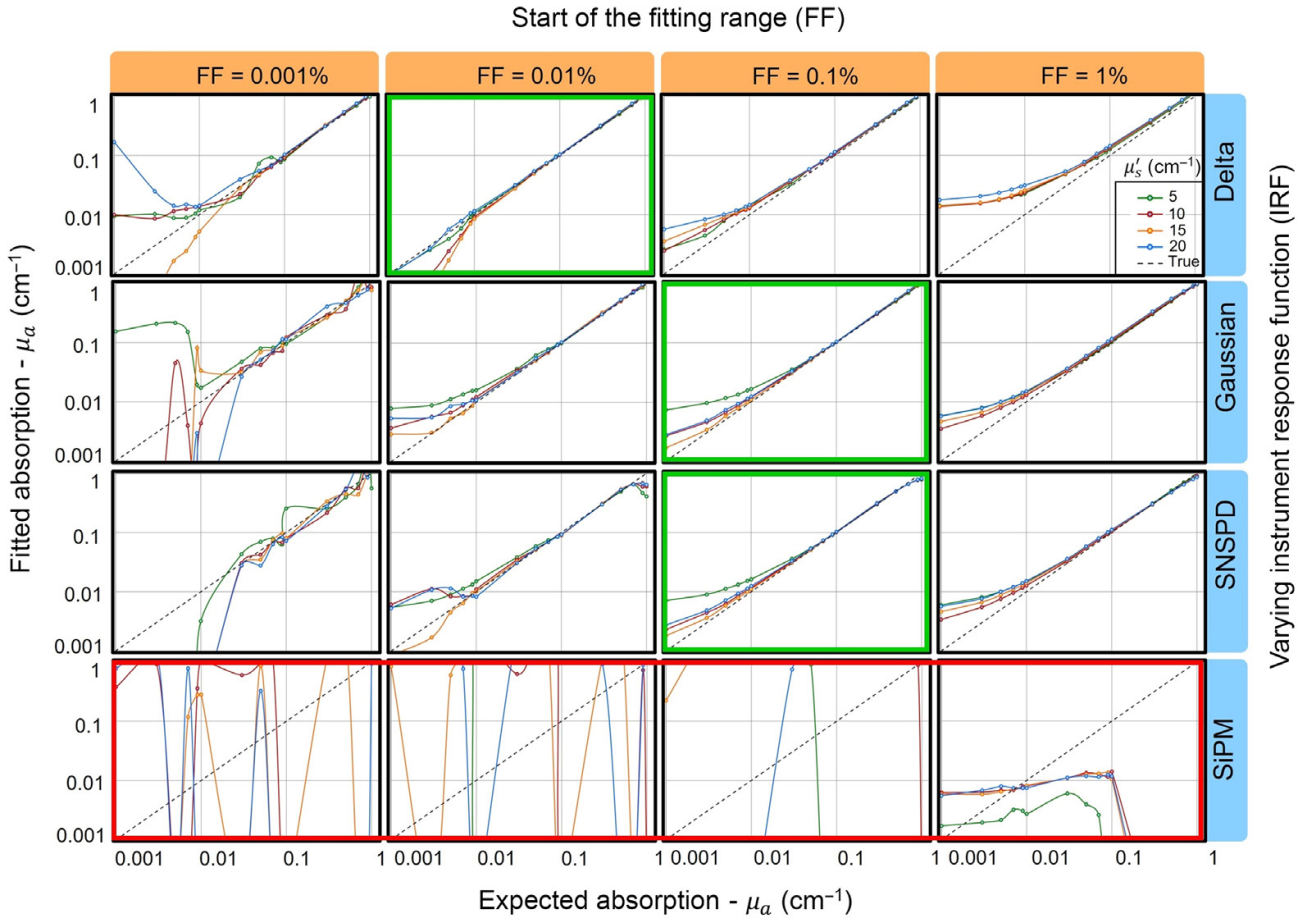
Linearity test of fitted versus expected absorption with MC simulations. The rows contain absorption linearity spectra from DTOFs convolved with different IRFs, and the columns change as the start of the fitting range on the falling edge of the DTOF. Colored frames represent the best (green) and worst (red) cases.

The other point to be noted is the negligible effect of varying $\mu_{s}^{\prime }$ on the DTOFs. In the case of the DE, this is expected because, for ρ=0 the only dependence on $\mu_{s}^{\prime }$ is an amplitude factor, which vanishes after normalization to compensate for the unknown optical responsivity (as discussed previously), and for a given $\mu_{a}$, one observes no change in the shape of the DTOF curves upon changing $\mu_{s}^{\prime }$. In the case of the MC simulations, there is a slight, yet negligible variation in the slope, which again suggests that keeping ρ=0 one could possibly remove $\mu_{s}^{\prime }$ in the fitting process and still retrieve $\mu_{a}$ accurately. Integrating these two observations into our analytical approach, we fitted the DTOFs obtained from MCX using in-house developed software. The effect of variation of $\mu_{s}^{\prime }$, IRF, and fitting range is summarized in Fig. 4.

The major observation is the high dependence on the fitting range and the extremely low value of the beginning of the fitting range (expressed as a fraction of the peak value). We observe optimized fitting ranges to begin at +0.05% to +0.1% on the falling edge, whereas in TD-DOS experiments at typical values of ρ=2  cm, a fitting range from −80% on the rising edge is usually adopted.^34^ This implies that the entire peak is excluded from the fit, which then depends only on the tail of the curve. Consequently, as anticipated, the results obtained from the SiPM are very poor, which can attributed to the long diffusion tail of the IRF that reduces the dynamic range of the detector, causing a disruption of fluence information from the late photons^31^ due to the overwhelming long-range contamination of early photons. In comparison, the delta, Gaussian, and SNSPD IRF convolved DTOFs yield good results with a relative error of 2% to 20% in selected fitting regions for absorption in the 0.01–1 ${\mathrm{cm}}^{-1}$ range.

One other point of interest is the mismatch in the optimized fitting range between the delta function and the other IRFs. Results for the delta IRF are the worst for many fitting ranges, and generally a lower threshold on the trailing edge is needed to preserve small errors. This is reasonable because, for non-delta like IRFs, the peak value is smoothed out upon convolution with the IRF, causing the region of independence from the burst of early photons to be reached at a lower peak fraction.^24^^,^^39^ In practice, the start of the error-free fitting range is almost the same for all “good” IRFs when expressed in TOF (data not shown).

As anticipated from the discussion in the theory section, the effect of $\mu_{s}^{\prime }$ is overshadowed by the exponential decay effect due to absorption, and variation of $\mu_{s}^{\prime }$ of the medium does not affect the $\mu_{a}$ retrieval significantly as the difference in relative errors is negligible. This is why, at higher absorption (0.03 to $1\;{\mathrm{cm}}^{-1}$), we observe relative errors of 2% to 10%, whereas at lower absorption (0.001 to $0.02\;{\mathrm{cm}}^{-1}$), where it dominates less, the relative error rises above 20%. However, through the entire range, the deviation from the mean relative error upon varying $\mu_{s}^{\prime }$ still remains very small. The only exception is at $\mu_{s}^{\prime }=5\;{\mathrm{cm}}^{-1}$, where we observe a large overestimation of $\mu_{a}$ at a lower absorption, which is anticipated due to the breakdown of the DE at lower scattering values.

**Fig. 5:**
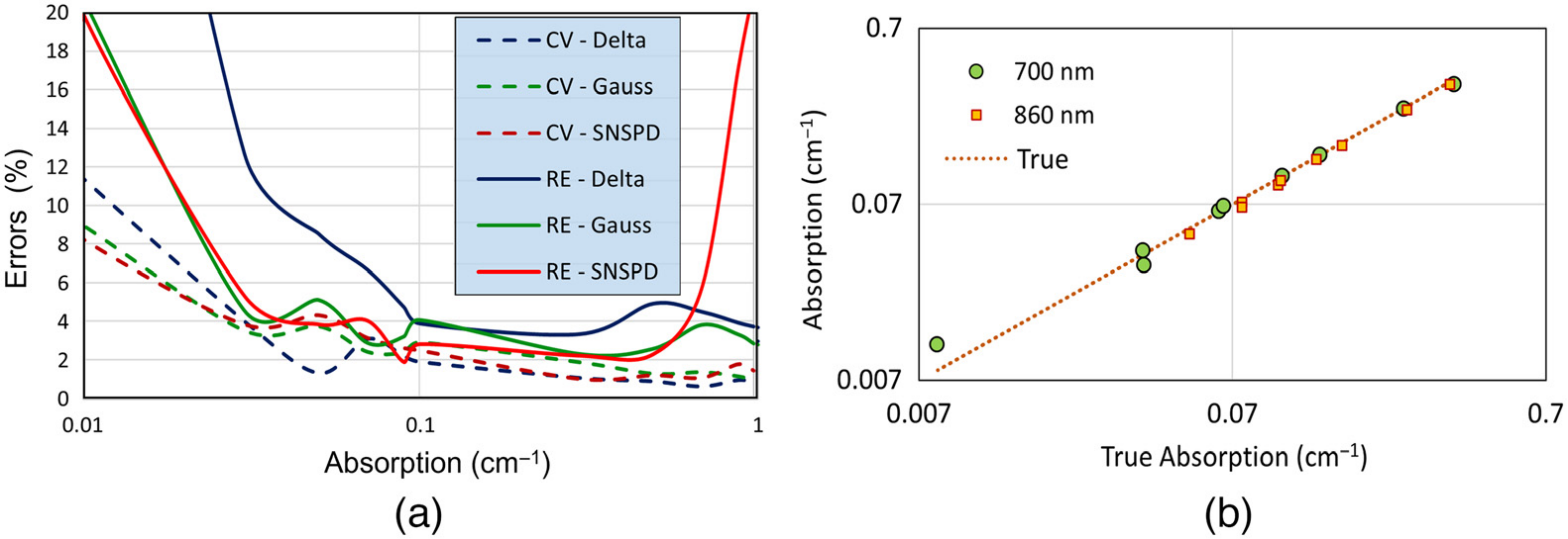
(a) Comparision of errors arising due to different IRFs. CV, coefficient of variation; RE, relative error. (b) Linearity measurements with the SNSPD at 700 and 860 nm.

Figure 5(a) displays the uncertainty in the simulations (dashed curves) calculated as the coefficient of variation (CV) among 10 different MC runs with different seeds, with the solid lines representing the relative error. For an initial input of $10^{6}$ photons, the CV was found to be less than 4% in the range of 0.03 to $1\;{\mathrm{cm}}^{-1}$ and rose up to 20% in the lower absorption range, showing that the observed error is not arising just from the uncertainties in the simulations.

## 5 Phantom Measurements

To assess the linearity of the system in retrieving the absorption coefficient, we performed a series of measurements on IL solutions with eight additions of ink corresponding to a 12-fold rise in ink concentration, with an expected rise in the absorption coefficient of about two orders of magnitude. For the analysis, we followed the same procedure as discussed in Sec. 4 and attempted to recover $\mu_{a}$ independent of $\mu_{s}^{\prime }$. Hence, we could test only the absorption linearity of the system. Given the dimensions of the box, the maximum distance from the black box boundaries are 30 mm on the Z axis, 35 mm on the Y axis and 50 mm on the X axis. By restricting our fitting range to <2  ns in this case, we can avoid the effect of the boundaries. Figure 5(b) displays the corresponding linearity plot at two different wavelengths. Experimental data for different series of absorption values (dots) lie on the expected linear trend (line). The results show that the system is linear in recovering $\mu_{a}$ values, with <10% relative error in the absorption range of 0.008 to $0.35\;{\mathrm{cm}}^{-1}$. This is the case at other wavelengths too (not shown), and the only discrepancy is a marginal underestimation around 960 to 980 nm, which represents the high absorption region of water, also commonly referred to as the “water peak.”

**Fig. 6:**
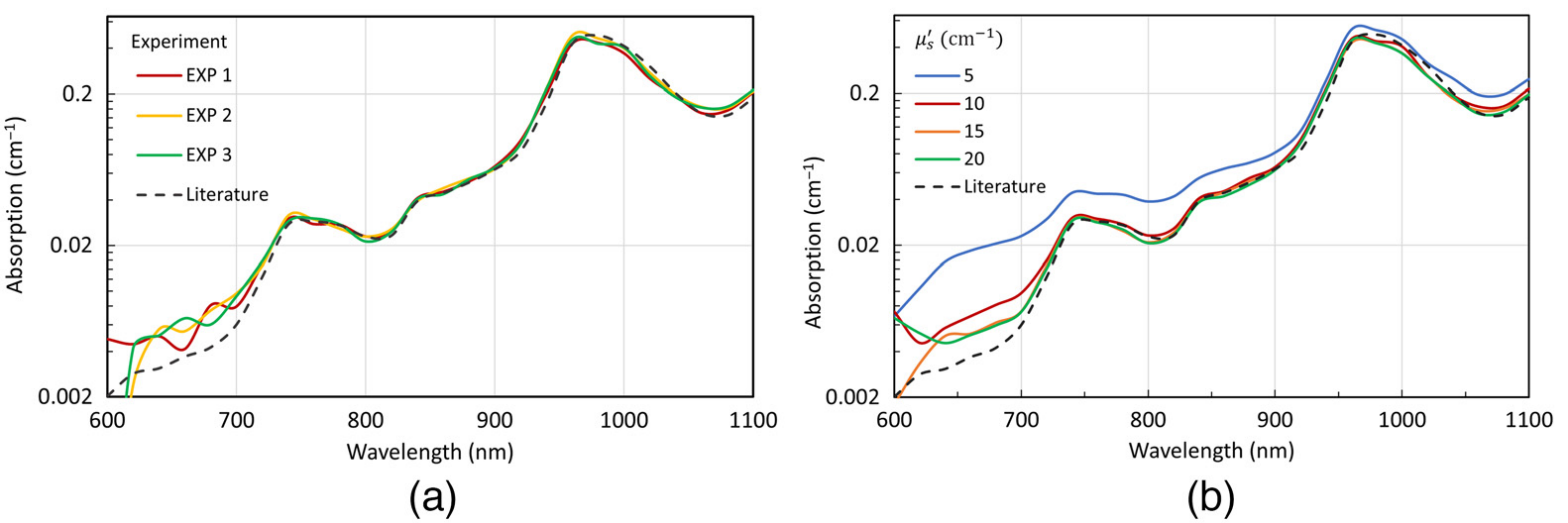
(a) Absorption spectrum of water obtained from three different experiments from liquid phantoms with $\mu_{s}^{\prime }=10\;{\mathrm{cm}}^{-1}$. (b) Effect of scattering on $\mu_{a}$ retrieval, where different curves represent different $\mu_{s}^{\prime }$ values (${\mathrm{cm}}^{-1}$).

Figure 6(a) shows the measured absorption spectra for three repetitions on a water + IL phantom with $\mu_{s}^{\prime }=10\;{\mathrm{cm}}^{-1}$ and without the addition of ink compared with the absorption spectra of water from the literature. The fitted $\mu_{a}$ values correspond very well to the expected values, well within 12% for almost the entire wavelength range. The discrepancies in the 600–700 nm range, where the relative error rises to about 50%, could be attributed to various factors: (i) low absolute values of $\mu_{a}(\approx 10^{-3}\;{\mathrm{cm}}^{-1})$; (ii) effect of BCs in the assumed “infinite” medium; and (iii) residual absorption by dispersed lipid molecules compared with the absorption spectrum of pure water.

Figure 6(b) also shows the effect of $\mu_{s}^{\prime }$ on the retrieval of water absorption spectra. As anticipated from the simulations, its effect is negligible, with less than a 5% deviation in relative error upon changing the scattering of the medium for $\mu_{s}^{\prime }=10\;{\mathrm{cm}}^{-1}$ or more (nominal values at 750 nm). The outlier from this trend is $\mu_{s}^{\prime }=5\;{\mathrm{cm}}^{-1}$, which shows quite a bit of overestimation, but this deviation was already anticipated from the MC simulations.

## 6 Conclusions

TD-DOS offers the advantages of amplitude independence, disentanglement of optical properties, and greater depth information. In addition, measurements at NSDS offer better contrast, greater spatial resolution, and higher signal, but they suffer from the drawback of detector saturation due to the burst of early photons.^22^ Usually, an ultrafast gating is applied to the detector to solve this problem, but again it has certain complications. By utilizing an SNSPD with a high dynamic range (>55  dB) and low noise characteristics, we have demonstrated the use of broadband TD-DOS in a NSDS approach without any gating. We performed MC simulations and studied the effect of convolution with different IRFs, noting the role played by a long tail in losing information from late photons. The importance of the fitting range and the independence from scattering were also studied. Further, from experiments performed on water + IL based liquid phantoms, by removing $\mu_{s}^{\prime }$ from the inversion process and fitting only late photons, we were able to recover the absorption spectrum of water with <15% relative error in the 700–1100 nm range. We verified the linearity of the system in the absorption range of 0.008 to $0.4\;{\mathrm{cm}}^{-1}$ and the independence of $\mu_{s}^{\prime }$, confirming the agreement between simulations and experiments. The SNSPD has certain drawbacks that include its high cost and its complicated setup including a cryostat under vacuum operation and a compressor for maintaining the liquid helium, both of which are limitations for a clinical system. However, this is an emerging field with research progressing rapidly, and this makes the SNSPD a viable option and a good solution for future research in needle guided time-domain IFS.
